# Advances in Molecular Dynamics-Based Characterization of Water and Ion Adsorption and Transport in C-S-H Gels

**DOI:** 10.3390/polym16233285

**Published:** 2024-11-25

**Authors:** Xinjie Li, Yingfang Fan, Chang Wu, Lei Wang

**Affiliations:** Institute of Road and Bridge Engineering, Dalian Maritime University, Dalian 116026, China

**Keywords:** calcium silicate hydrate, molecular dynamics, water molecules and ions, transport and adsorption properties

## Abstract

Cementitious material durability is affected by the transport and adsorption of water molecules and ions in the nanopore channels of cement hydration products. Hydrated calcium silicate (C-S-H) accounts for about 70% of the hydration product. It determines the mechanical properties of cementitious materials and their internal transport properties. The molecular dynamics method provides a complementary understanding of experimental and theoretical results. It can further reveal water molecules and ions’ adsorption and transport mechanisms in C-S-H gel pores. This review article provides an overview of the current state of research on the structure of C-S-H gels and the adsorption and transport properties of water molecules and ions within C-S-H gels, as studied through molecular dynamics simulations. This paper summarizes the results of the molecular dynamics-based adsorption transport properties of water molecules and ions in C-S-H gels. The deficiencies in the current study were analyzed, and the fundamental problems to be solved and further research directions were clarified to provide scientific references for revealing the structural properties of C-S-H gels using molecular dynamics.

## 1. Introduction

The civil engineering community faces the engineering challenge of the durability of cementitious materials in hazardous media environments. Cementitious materials are porous, and many defects and pores inevitably exist. These provide a conduit for transporting harmful agents within the concrete, seriously affecting the durability of the cementitious material itself and reinforcement.

Hydrate calcium silicate (C-S-H) gels account for about 70% of the hydration products of cement. They are the primary source of strength of cementitious materials, and determine the materials’ structure and properties [1]. Numerous microscopic pores (0.5–10 nm) existed inside the C-S-H gel structure, which will form a nanopore channel. On the one hand, it provides sites for transporting water molecules and ions in the C-S-H gel structure in the service environment. On the other hand, it provides conditions for physicochemical reactions of ions near the surface of hydration products. As a result, the durability of cementitious materials will be directly affected. Compared to porous material adsorbents with regular pores and tunable chemical functionality, such as metal–organic frameworks (MOFs) and covalent organic frameworks (COFs) [2,3], C-S-H gel possesses unique advantages in adsorption performance due to its nanoscale disordered microporous structure and high specific surface area. Similar to MOFs [4], the interactions between C-S-H gel and adsorbates encompass both physical adsorption and chemical adsorption, which are significantly influenced by factors such as pH value and the calcium-to-silica ratio. In metro projects or tunnel projects, erosive ions adsorbed into the matrix undergo a complex physicochemical reaction, leading to concrete deterioration [5]. With the development of nanotechnology, the characterization and enhancement of the properties of cementitious materials have been gradually extended to the micro- and nanoscale. With the help of molecular dynamics methods, discussing the C-S-H structure and properties from the molecular and nanoscale is possible. It can reveal its microscopic mechanism, ensure simulation accuracy, and explain macroscopic experimental phenomena. C-S-H gel is a composite layered structure composed of calcium sheets and silicate chains [6]. The silicate tetrahedra that are directly connected to each other are referred to as paired, while those connecting two paired tetrahedra are known as bridging tetrahedra. Additionally, water molecules, hydroxyl groups, and interlayer calcium ions are primarily found near the surface of the bridging tetrahedra or in the channels between adjacent bridging tetrahedra. This structure endows C-S-H gels with unique chemical and physical properties.

Several scholars have summarized and reviewed the molecular dynamics studies on the structural model, mechanical properties, structural parameters (Ca/Si ratio, water content, degree of polymerization, and hardness) and their characterization methods of C-S-H gels, internal water molecule migration micro-mechanisms, and drying shrinkage of cementitious materials [7,8,9,10]. However, studies involving the adsorption and transport processes of water molecules and ions in C-S-H gels based on molecular dynamics methods have not been reported in reviews.

This article focuses on water molecules and ions’ adsorption and transport characteristics in C-S-H gel. It systematically introduces the establishment of C-S-H gel models and the selection of force fields. The development of the C-S-H gel model and the choice of molecular models are highlighted. The adsorption and transport properties of water molecules and ions in C-S-H gels have also been particularly emphasized. This paper reveals the mechanism of water and ions’ effect on cementitious materials’ properties. This article innovatively compiles research on the adsorption and transport characteristics of water molecules and ions in C-S-H gels at the molecular scale, which holds significant theoretical value and engineering importance for elucidating the processes of structural deterioration and the assessment of durability.

## 2. Molecular Modeling of C-S-H Gels

### 2.1. Development of Structural Models

C-S-H gels are amorphous substances containing a variety of components. Due to their composition and structure complexity, their structure is challenging to standardize. Previous studies have investigated the structure of C-S-H gels at various scales and modeled its microstructure. However, due to their complexity, a wide variety of C-S-H gel microstructure models are available.

The study of microstructural modeling of C-S-H gels has been going on consistently since the 1950s. Before the development of observation and characterization techniques, nanoscale C-S-H gel structure models were developed [11]. These models explain the structural parameters and physical properties, such as specific surface area, density, and volume deformation of the C-S-H gel structure. Among them, the classical models mainly contain the P–B model [12], the F–S model [13], and the Munich model [14]. Among them, the P–B model is a gel-like microscopic model. It consists of 2–3 layers with a spacing of 1.4 nm formed by 10 nm diameter particles. The F–S model is based on the P–B model. It is considered that the nanoscale C-S-H gel is a clay-like layered microcrystal with an irregular arrangement of layered particles. Moreover, the P–B and F–S models represent two predominant schools regarding the nanoscale structure of C-S-H: the colloidal model (e.g., the P–B model) and the continuous model (e.g., the F–S model) [15,16]. In contrast, the Munich model defines a C-S-H gel as an amorphous structure. These models provide a foundation and methodology for subsequent research, offering an understanding of the structural characteristics of C-S-H gel at the nanoscale, including its layered structure, pore distribution, and the adsorption state of water molecules. Furthermore, parameters within the models such as the calcium-to-silica ratio, interlayer spacing, and water molecule content provide a basis for the initial parameter settings in molecular dynamics simulations. However, due to the lack of morphological research, the description of the models remains in the theoretical stage.

Furthermore, the CM model has been expanded upon the basis of the P–B model, which describes the aggregation patterns of C-S-H particles. The CM model is a colloid (nanostructure model) built based on the structural data provided by the R–G model. Jennings and Tennis first proposed the CM-I model [17], in which C-S-H gels are composed of close-packed micelles approximating the smallest structural unit of a spherical body. According to the stacking density, they are classified into high-density hydrated calcium silicate (HD C-S-H) and low-density hydrated calcium silicate (LD C-S-H). Among them, LD C-S-H gels consist of calcium silicate flakes randomly connected through strong ionic covalent bonds. Subsequently, Jenning [18] proposed a laminar CM-II model with a modification of CM-I, stating that the smallest structural unit micelle is a spherical body with a diameter of less than 5 nm. Currently, MD simulations predominantly use a colloidal approach based on the CM-II model.

With the development of technology, scholars began to observe and recognize the C-S-H gel structure in terms of morphology. Jennings et al. [19] used UHV and transmission electron microscopy to observe C-S-H gels in hydrated tricalcium silicate (C_3_S) slurries. They established a link between the hydration reaction of cement at various stages and the morphological characteristics of C-S-H gels. Different assembly and coalescence modes between C-S-H gel nanoparticles give rise to different morphologies. Richardson [20] used transmission electron microscopy to observe that the external product of the C-S-H gel was fibrous and consisted of a bunch of long and thin particles with a minimum size of 3 nm combined. The C-S-H gel inner products consisted of 4–8 nm granular forms. At the same time, Nonat et al. [21] found that the basic structure of C-S-H gels is 60 × 30 × 5 nm^3^ brick-shaped nanoflakes and the flakes tend to agglomerate directionally when stacked. Scrivener et al. [22] found that the C-S-H gel morphology is essentially foil-like and that the needle-like C-S-H gel consists of foil-like C-S-H gel. The development of nano-characterization techniques has improved the understanding of the morphological characteristics of C-S-H gels. However, due to the amorphous and complex structure of C-S-H gels, there are many inconsistent conclusions on issues such as morphology, size, and composition.

The structure and properties of C-S-H gels show significant differences due to the different Ca/Si ratios and average silica chain lengths. The atomic-scale (<1 nm) structure of C-S-H primarily encompasses the Taylor model, the solid solution model, and the R–G model, which focus on the fundamental atomic structure of C-S-H particles. The Taylor model [23] was built based on the microstructure of Tobermorite and Jennite. The model categorizes the structures into two types: low Ca/Si ratio C-S-H gels (C-S-H(I)) and high Ca/Si ratio C-S-H gels (C-S-H(II)). Of these, C-S-H(I) accounts for only a tiny fraction. It is similar to the structure of defective Tobermorite-14Å (Ca_5_Si_6_O_26_H_18_) atoms and has a Ca/Si ratio of less than 1.5. C-S-H(II) is in the majority, similar to the atomic structure of defective Jennite(Ca_9_Si_6_O_32_H_22_), with Ca/Si ratios between 1.5 and 2. In the C-S-H(I) model, elemental silicon exists through a silica–oxygen tetrahedral chain structure. The twisted chain structures in them can tilt, break, etc., forming dimers and thus also appearing fragmented. The solid solution model [24] describes C-S-H gels as solid solutions of Tobermorite and calcium hydroxide (Ca(OH)_2_). Their sandwich-like layered structure of Tobermorite is sandwiched between two Ca(OH)_2_ layers. Richardson and Groves proposed the R–G model based on the solid solution model. C-S-H gels are considered solid solutions of combined Tobermorite, Jennite, and Ca(OH)_2_ [25]. It categorizes the C-S-H gel models into the T/J model with Tobermorite and Jennite structural composites and the T/CH model with Tobermorite and Ca(OH)_2_ composites. This model is considered highly versatile as it encompasses not only the properties and relative proportions of ions but also their specific arrangements [26]. Richardson [27] modified the model by studying the adsorption of metal ions on C-S-H gels. It is thought that aluminum ions and the like can displace silicon on the silica–oxygen tetrahedra and that silicon binds with interlayer calcium ions or other alkali metal ions to neutralize the resulting charge imbalance. A comparative and analytical review of three C-S-H gel structural models in terms of theoretical basis, structural morphology, and application scope is detailed in Table 1. The three models have different advantages and disadvantages as well as applications, and the Taylor model successfully explains the characteristic of C-S-H having a disordered layered structure and is one of the most widely used models [28,29]. The solid solution model has been used extensively to study, among other things, cement hydration. Still, the model does not consider the effect of the calcium/silicon ratio on the structure of C-S-H gels. The R–G model can evaluate the C-S-H gel structure in new and old cementitious materials. These three models all attempt to explain the structure and properties of C-S-H at the atomic level, but they emphasize different aspects. The Taylor model highlights the nanoscale structure and porosity of C-S-H, and the R–G model focuses more on the layered structure and distribution of calcium ions in C-S-H, while the solid solution model emphasizes the continuous variation in the chemical composition of C-S-H.

Some differences between the above models confirm the complexity of the C-S-H gel system from the side. In the study of C-S-H gels, it is necessary to determine the use of the model according to the purpose, and the construction of a reasonable, universal, and accurate structural model of C-S-H gels needs to be continuously explored.

### 2.2. Molecular Structure Model

Molecular dynamics is a method of modeling matter from the atomic scale. Therefore, studying C-S-H gels from a microscopic point of view requires determining a molecular model composed of atoms. C-S-H gels are essentially amorphous complex structures. Thus, when studying it microscopically, it is impossible to accurately, graphically, and uniformly model C-S-H gels. In current molecular dynamics studies of C-S-H gels, Tobermorite and Jennite structures, which are similar to C-S-H gels [9], are generally used as the basis for constructing molecular structure models of C-S-H gels. The lattice parameters of Tobermorite and Jennite are presented in Table 2, with data sourced from the Crystallography Open Database. The interlayer spacings of the Tobermorite structure include 0.9 nm, 1.1 nm, and 1.4 nm, corresponding to Tobermorite-9Å, Tobermorite-11Å, and Tobermorite-14Å, respectively.

Jennite is a mineral with the structural chemical formula Ca_9_Si_6_O_32_H_22_. Bonaccorsi et al. [30] proposed the Jennite crystal model using single-crystal X-ray Diffraction (XRD) analysis. The model is similar to the Taylor model with a triclinic crystal system. The crystal structure consists of three parts: calcium-oxygen octahedral banding, specially positioned calcium-oxygen octahedra, and Dreierketten-type silicon chains. Subsequently, Sergey et al. [31] used ab initio molecular dynamics methods to analyze the Jennite hydrogen bonding structure based on the structure proposed by Bonaccorsi. The Jennite crystal structure has a calcium-to-silicon ratio of about 1.5, higher than the Tobermorite crystal structure. It is more similar to the actual state of C-S-H gel.

At the atomic level, there are two well-known models for Tobermorite-11Å, such as the Hamid model [32] and the Merlino model [33]. The main difference between the two is the presence or absence of chemical bonds between the silicate chains and the layers of calcium atoms. The layer-to-layer interaction in the Hamid model relies mainly on the formation of bridge-site silica–oxygen tetrahedra and the Coulombic interaction of interlayer Ca^2+^. The layers are not chemically bonded and are independent of each other. The Merlino model relies mainly on the covalent bonds formed between the bridge-site silica–oxygen tetrahedra to enhance the layer-to-layer interaction.

**Figure 1:**
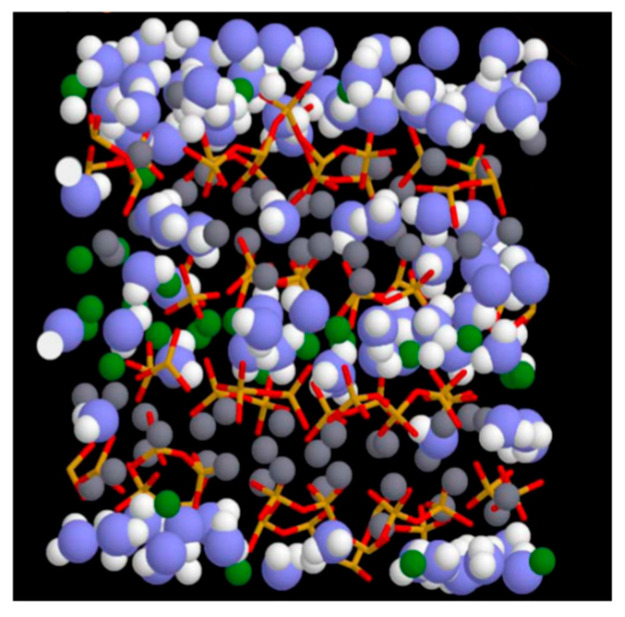
The “realistic model” proposed by Pellenq: the blue and white spheres are oxygen and hydrogen atoms of water molecules, respectively; the green and gray spheres are inter- and intralayer calcium ions, respectively; yellow and red sticks are silicon and oxygen atoms in silica tetrahedra [34].

Pellenq et al. [34], utilizing precise experimental data, constructed a molecular structure model for amorphous C-S-H gels based on anhydrous Tobermorite-11Å with the chemical formula (CaO)_1.65_(SiO_2_)(H_2_O)_1.75_. They employed regular Monte Carlo simulation methods to model the adsorption of water in defective C-S-H, achieving a reasonable chemical composition and density. The model has a density of 2.6 g/cm^3^. By reducing the bridging silicate tetrahedra, they adjusted the calcium-to-silica ratio to 1.65 and the water-to-silica ratio to 1.75. Through the aforementioned methods, they obtained the molecular structure model of amorphous C-S-H gels, referred to as the “real model”. It was proposed based on accurate data from experimental measurements and the application of molecular dynamics and the Monte Carlo method. The model density was 2.6 g/cm^3^, and the calcium/silica ratio was adjusted to 1.65 and the water/silica ratio to 1.75 by deleting the bridging silica–oxygen tetrahedra. The model is the first nano-model established by molecular simulation that characterizes the short-range ordering of the glass-like structure of C-S-H gels. It fully reflects the characteristics of silicon chains with defects [35], which can be well-matched with authentic C-S-H gels regarding mechanical characteristics, average density, and chemical composition. Figure 1 represents the basic structure of the model; it is now widely used in molecular modeling studies of C-S-H gel adsorption and transport, mechanical properties, thermodynamics, reactivity, and other properties [36,37]. The Pellenq model provides structural characteristics of C-S-H gels at the atomic scale, including their layered structure, pore distribution, and the adsorption state of water molecules, which offers a more accurate initial structural model for molecular dynamics simulations. However, the model also has some limitations, and scholars usually use the modeling method of the model to construct more reasonable C-S-H gel structures. Another influential model is the brick model proposed by Kunhi Mohamed [38], which takes into account various defects, such as the replacement of bridging silicate tetrahedra by calcium ions and the replacement of silicate dimers by hydroxyl groups, represented by specific symbols in the model. It provides a detailed description of the atomic-scale structure of C-S-H, including the Ca/Si ratio, the length distribution of silicate chains, the number of interlayer hydroxyls, and so on. These characteristics are crucial for explaining the physical and chemical behavior of C-S-H gels. Furthermore, the model can predict the thermodynamic behavior of C-S-H gels under different temperatures and pressures, which is of great significance for the design and application of cement-based materials.

### 2.3. Molecular Simulation of C-S-H Gel

#### 2.3.1. Selection of Molecular Force Fields

In molecular dynamics simulation, the choice of molecular force field is directly related to the accuracy and rationality of the calculation results of molecular dynamics simulation. Therefore, in establishing molecular modeling, choosing a suitable molecular force field to describe the interatomic interactions is necessary. The molecular force field uses a mathematical analytic formulation of the classical potential function to describe the intermolecular interaction potential. It consists of three parts: atom type, possible function, and force field parameters. To date, a force field is lacking that uniformly describes all systems in molecular dynamics simulations of cementitious materials. The molecular force fields used by different scholars have their advantages.

For the simulation of calcium silicate systems, force fields such as COMPASS [39], ClayFF [40], CSH-FF [41], Reactive Force Field (ReaxFF) [42], CementFF [43], INTERFACE [44], and CemFF [45] are generally used. COMPASS force fields are high-quality generalized ab initio arithmetic force fields widely used in liquid, crystal, and polymer simulations. It can simulate the mechanical properties of C-S-H gels, interlayer ion diffusion and adsorption. The ClayFF force field can simulate water and multi-component mineral systems, aqueous solution interfaces, calcium silicate systems, and large and highly disordered systems [46]. It uses the classical Simply Point Charge (SPC) force field to describe water molecules. It has been widely used in molecular dynamics studies of C-S-H gel mechanics, adsorption, and transport properties [1,47,48,49,50,51]. By improving the parameters of Ca, Si, and O in the ClayFF force field, the CSH-FF force field proposed by Shahsavari et al. [41] more accurately predicts the elastic modulus and structural data. Its calculations of the structural and elastic properties of Tobermorite-14Å agree with the results of the nanoindentation tests. This force field can correctly predict the essential structural and physical characteristics of cement hydrates. It is now widely used in molecular simulations of the mechanical and thermodynamic properties of C-S-H gels [52,53,54]. The ReaxFF force field describes the interactions between Ca, Si, O, and H atoms in C-S-H gels. It can simulate chemical reaction processes, including chemical bond formation and breaking. This force field is widely used in inorganic systems such as silicon–water interfaces, C-S-H gels, and clay minerals [6,55,56]. The INTERFACE force field is typically utilized for simulating the assembly of inorganic, organic, and biological nanostructures, with a particular emphasis on atomic charges and van der Waals parameters with chemical significance. While this force field encounters challenges in simulating chemical reactions, it can incorporate specific algorithms for chemical reactions. The CementFF force field is suitable for cementitious systems, capable of capturing lattice parameters and deriving elastic properties from elastic tensors, with deviations from experimental values falling within an acceptable range. The Cemff force field database is an online resource designed to provide force field parametrization, validation, application, and opportunities for the molecular simulation of cement-based materials. This database offers information on various force fields, energy expressions, and model validations by systematically comparing computational data with experimental benchmarks and ab initio calculations. The strength of this force field database lies in its comparative analysis of different force field models, assisting in the selection of the most appropriate simulation technique to address specific atomic-scale issues. Common simple water molecule force field models include simple point charge (SPC), extended simple point charge(SPC/E), and the three point (TIP3P) and four point (TIP4P) transferable intermolecular potentials [57]. These models essentially adopt a rigid water molecule structure, that is, with fixed bond lengths and bond angles. The potential field between water molecules is described through van der Waals interactions and electrostatic interactions. In the selection of force fields, the parameterization’s overarching structure must be simplistic enough to facilitate the modeling of highly disordered systems comprising a substantial number of atoms, while effectively capturing their intricate and frequently cooperative behaviors. Studies have indicated that the aforementioned force fields exhibit varying degrees of applicability in describing the structure and properties of C-S-H gels. The majority of water models in simulations utilize the SPC model, as it offers accuracy and computational efficiency in simulating cement-based materials at the molecular scale [58]. Currently, due to the accuracy and applicability of ClayFF, it is widely adopted to simulate the entire process [1].

#### 2.3.2. Boundary Condition

Common model boundaries utilized in molecular dynamics simulations include the Fixed Boundary Condition (FBC) and the Periodic Boundary Condition (PBC). The FBC prevents atoms from passing through, effectively confining them within a designated region. Consequently, the FBC is suitable for simulating the microscopic behavior of a small number of molecules within a restricted space. Moreover, the interaction between atoms and the boundary must be carefully considered when employing the FBC. If the objective of the study is to investigate the macroscopic properties of a system or a local part of a macroscopic real system, the PBC is typically applied in at least one dimension. The PBC facilitates the study of various properties of a material by simulating a relatively small number of atoms. The size of the simulation box has a significant impact on both the accuracy of the simulation and the computational time required. A larger box implies a greater number of atoms, which in turn demands more computational resources and time. In molecular dynamics studies involving the transport and adsorption of water molecules and ions within C-S-H gels, periodic boundary conditions are generally adopted.

#### 2.3.3. Ensemble

In molecular dynamics simulations of multi-particle systems, the number of particles is finite, yet the statistical physical laws hold true, necessitating the selection of an appropriate system. An ensemble refers to a collection of systems that are structurally identical and subject to given macroscopic conditions. Particles within an ensemble do not interfere with each other and are in their respective states of motion. The main ensembles include the isothermal–isobaric ensemble (NPT), the canonical ensemble (NVT), the microcanonical ensemble (NVE), and the isothermal–isenthalpic ensemble (NPH), among others. In studies on the transport and adsorption of water and ions in C-S-H gels, the migration of ions and physical adsorption may vary over time depending on the ensemble used, and papers generally select different ensembles based on specific circumstances. For instance, reference [1] employs the NPT ensemble when constructing models, and the NVT ensemble for quasi-adsorption quantity calculations.

## 3. Transport and Adsorption of Water Molecules and Ions

The durability degradation of cement-based materials due to sulfate or chloride attack and creep is inseparable from the involvement of water molecules [53,59]. The adverse effects of erosive ions, such as the induction of steel reinforcement corrosion and the deterioration of cement-based materials, are closely related to the adsorption and transport characteristics of water molecules and ions within the C-S-H gels. Therefore, understanding the adsorption and transport processes and mechanisms of water molecules and ions within the nano-pores of C-S-H gels can effectively inform measures to prevent the degradation of cement-based materials. Currently, some experimental and theoretical analyses only explain the mechanisms at a macroscopic level. Molecular dynamics methods can provide a more profound insight at the nanoscale.

### 3.1. Adsorption of Water Molecules in C-S-H Gels

**Figure 2:**
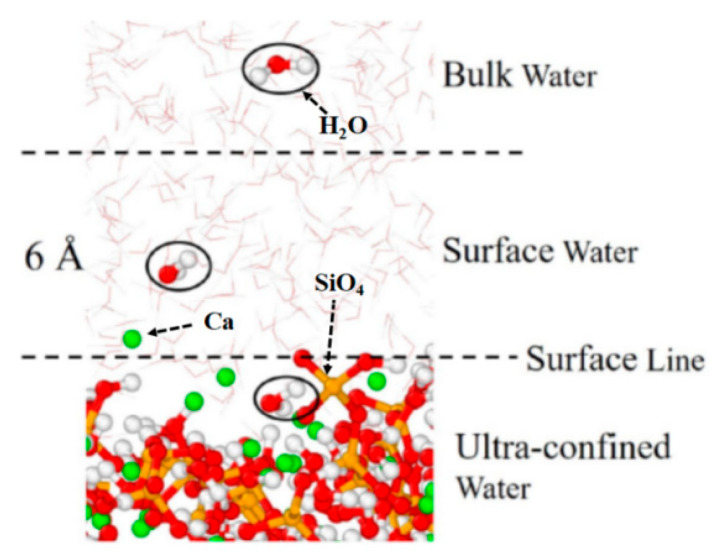
Classification of water molecules in C-S-H gels [60].

Water molecules in C-S-H gels are categorized based on the location of oxygen atoms: interlayer water molecules, surface water molecules, and capillary water molecules. Interlayer water molecules are located within the C-S-H gel matrix, surface water molecules are within 6 Å of the outer surface of the matrix, and capillary water molecules are outside the C-S-H gels (Figure 2) [60]. Compared to capillary water, surface and interlayer water molecules exhibit more ordered structures, higher densities, greater orientational mobility, and lower diffusion coefficients [61].

The orderly stacking of water molecules in C-S-H gels is attributed to the interactions between water molecules and hydrophilic groups on the surface of C-S-H gels, including silicate chains, interlayer, and intralayer calcium ions [62]. By utilizing molecular dynamics methods, some scholars have studied the hydrophilicity of the crystal structure of C-S-H gels [62,63]. As the solution diffuses, the decreasing contact angle between the solution and the surface of the C-S-H gel reflects the hydrophilicity of the gel’s surface. Water molecules within the silicate channels form ionic bonds with interlayer calcium ions, with hydroxyl groups pointing outwards. When water molecules approach the C-S-H gel interface, the surface calcium ions available for hydrogen bonding coordination attract the oxygen atoms in water molecules, forming stable Ca–O bonds that influence the orientation of water molecules. The lower diffusion rate of water molecules near hydrophobic surfaces is due to the ice-like water layer of silicate crystals, while the lower kinetic performance of water molecules near hydrophilic surfaces is due to strong hydrogen bonds in the interfacial region [64]. As the Ca/Si ratio increases, the silicate framework’s interstitial channels become narrower, making the water molecules adsorbed around the C-S-H gel interface more susceptible to the influence of outer water and ions, leading to a more complex spatial distribution. In C-S-H gels with a high Ca/Si, there is a reduction in bridging silicate tetrahedra and an increase in calcium ions, which results in a more pronounced stratification of water molecules [65].

Researchers have provided new insights into the microphysical properties of cement-based materials by simulating the transport characteristics of water molecules and ions on the surface of C-S-H. These studies have not only enhanced our understanding of the adsorption mechanisms of water molecules within C-S-H gels but also offer significant theoretical support for the development and improvement of cementitious structures.

### 3.2. Erosive Effect of Harmful Ions

The migration and physical adsorption of ions in C-S-H gels over time are challenging to capture using experimental methods. Currently, the adsorption capacity of cement-based materials can be assessed by analyzing the microstructure and thermodynamic modeling to determine the content of Friedel’s salt, thereby indirectly calculating the physical adsorption amount of Cl^−^ [66]. Or synthetically different Ca/Si ratios of C-S-H to simulate the state of C-S-H in cement at various ages, and measure the physical adsorption amount by zeta potential titration method [67]. However, the accuracy of indirect measurement methods is susceptible to the influence of external environments, and the hydration synthesis method cannot guarantee the purity of the synthesized samples [68]. At the same time, neither of these methods can directly display the changes in the migration rate of ions during the physical adsorption process in C-S-H gels.

In contrast, molecular dynamics simulation methods can provide reliable results for the migration dynamics characteristics of ions within molecular structures. In molecular dynamics simulations, a layered model is commonly used to explore the adsorption and transport of ions within C-S-H gel, with the pore sizes of C-S-H gel typically ranging from 0.5 to 10 nm [69]. For instance, literature [68] sets the pore width to 60 Å.

In deep geological or marine environments, the presence of Cl^−^ in the system is one of the main factors leading to the deterioration of load-bearing capacity and reduced durability in reinforced concrete structures. Once Cl^−^ enters the concrete, it primarily exists in two forms: free Cl^−^ and bound Cl^−^. When the concentration of free Cl^−^ reaches a critical value, the alkalinity of the passive film on the surface of the reinforcement decreases and is destroyed, causing rusting of the reinforcement [70]. The binding effect of cement hydration products on Cl^−^ can solidify a portion of the free Cl^−^, thereby delaying the time it takes to reach the rebar’s surface [71]. The binding effect of cement-based materials on Cl^−^ is typically divided into chemical binding and physical adsorption [72]. Chemical binding mainly refers to the formation of low-solubility Friedel’s salt (3CaO·Al_2_O_3_·CaCl_2_·10H_2_O) through the solidification of Cl^−^ with hydrated ferric aluminate (AFm), while unbound Cl^−^ continues to diffuse in the pore solution of the cement-based material in a free state. Physical adsorption mainly includes the electrostatic attraction and van der Waals forces that C-S-H gels exert on Cl^−^ through its surface charge. The adsorption capacity of C-S-H gels for Cl^−^ is relatively weak and susceptible to environmental influences and other ions, leading to desorption [71,72]. Since the content of C-S-H gel is much higher than that of AFm, physical adsorption is one of the critical states of Cl^−^ binding [73]. Molecular dynamics simulation studies on the interaction of Cl^−^ with cement have revealed the following order of chloride binding capacity: Friedel’s salt > Ca(OH)_2_ > ettringite (AFt) > Tobermorite, which is consistent with the results of ^35^Cl NMR experiments [74]. Research indicates that the adsorption of Cl^−^ by C-S-H gel is affected by factors such as the Ca/Si ratio, SO_4_^2−^, the type of action, and pH value.

To explore the impact of the Ca/Si ratio on Cl^−^ adsorption by C-S-H gel, molecular dynamics models of C-S-H gels with varying Ca/Si ratios (0.8, 1.2, 1.75) were constructed based on Pellenq’s “real model” [37]. At a Ca/Si ratio of 1.2, the C-S-H gel exhibited the highest Cl^−^ adsorption capacity. When the Ca/Si ratio is below 1.2, the adsorption amount and stability of Cl^−^ on the C-S-H gel surface increase with the rising Ca/Si ratio. This is attributed to a decrease in the proportion of Q^2^ species and an enhanced binding capacity of the silicate tetrahedra in C-S-H gels with cations. Conversely, when the Ca/Si ratio surpasses 1.2, the average chain length of C-S-H gel shortens, and the number of oligomers increases; the loose oligomers exhibit weaker Cl^−^ adsorption capabilities, leading to a reduction in Cl^−^ adsorption by the C-S-H gel. Thus, it can be seen that C-S-H gels with a low Ca/Si ratio can better impede Cl^−^ intrusion. C-S-H gels with a low Ca/Si ratio have a higher diffusion coefficient for Cl^−^, while those with a high Ca/Si ratio exhibit greater adsorption capacity for Cl^−^ [75]. In the presence of both high and low Ca/Si ratio C-S-H gel models, Cl^−^ tends to adsorb onto the surface of the high Ca/Si ratio C-S-H gel and stay away from the surface of the low Ca/Si ratio C-S-H gel [75,76].

Many scholars have used experimental methods, thermodynamic modeling, and molecular dynamics simulations to explore the erosion mechanisms of chloride-sulfate on cement-based materials [10,77,78,79,80,81,82] and have unanimously reached the following conclusions: (1) Sulfates reduce the binding capacity of hydration products for Cl^−^. SO_4_^2−^ reacts with AFm to form AFt, and since AFt is more stable than Friedel’s salt, the combination of SO_4_^2−^ with AFm leads to a decrease in AFm that is chemically bound with Cl^−^; on the other hand, SO_4_^2−^ can cause the decomposition of Friedel’s salt and eventually transform into AFt. (2) Sulfates reduce the physical adsorption of Cl^−^, that is, the binding capacity of C-S-H gel for Cl^−^. In NaCl + Na_2_SO_4_ solutions, Na^+^ tends to form large Na–SO_4_ clusters, thereby hindering the movement of Cl^−^, occupying the adsorption sites of Cl^−^, and thus reducing the binding capacity of C-S-H gel for Cl^−^. In addition, ion clusters also help in the secondary SO_4_^2−^ adsorption. (3) To some extent, chloride salts delay the sulfate erosion effect. Both Cl^−^ and SO_4_^2−^ can react with C_3_A and AFm, but Cl^−^ diffuses faster and enters the hardened cement paste earlier than SO_4_^2−^, delaying the production of AFt and weakening the sulfate erosion. However, using SO_4_^2−^ to inhibit the transport of Cl^−^ is effective initially, but it causes secondary damage to the concrete structure, reducing concrete strength. The performance changes and failure mechanisms of cement-based materials under the erosion of complex salt solutions differ significantly from those under single-factor actions and are not simply an additive effect of individual factors but involve complex coupled interactions.

**Figure 3:**
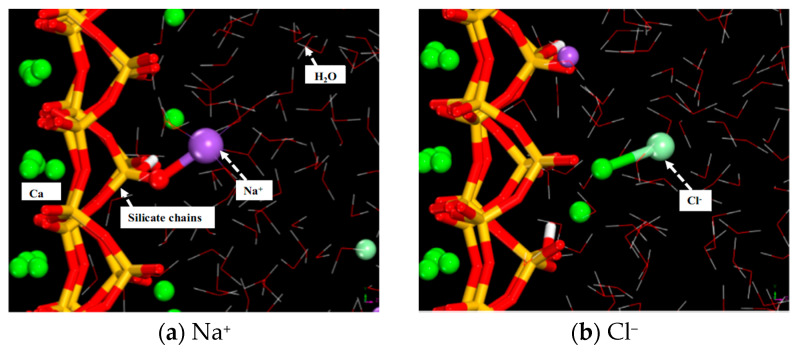
Local structures of (**a**) sodium ions and (**b**) chloride ions adsorbed on the C-S-H substrate [62].

Molecular dynamics studies on the impact of cation type on the adsorption of Cl^−^ by C-S-H gel have revealed that Ca^2+^ and Na^+^ enhance the surface adsorption capacity of C-S-H gel for Cl^−^ [37,76]. This is due to stable short-range Ca–Cl and Na–Cl clusters formed by Ca^2+^ and Na^+^ with Cl^−^. Under the same concentration, calcium salts more effectively promote the adsorption of Cl^−^ by C-S-H gel than sodium salts. In NaCl solutions, Na^+^ adsorbs onto the surface of the matrix due to the double-layer effect and coordinates with non-bridging oxygen atoms, adsorbing within the matrix (Figure 3a). A significantly higher amount of Na^+^ adsorbed on the surface of C-S-H gel than Cl^−^. The surface of C-S-H gel tends to attract Na^+^ preferentially, as the bridging oxygen atoms O_B_ and non-bridging oxygen atoms O_NB_ in the silicate tetrahedra carry a negative charge, attracting positively charged Na^+^.

In contrast, the negatively charged Cl^−^ is more distributed around Na^+^ and Ca^2+^. In molecular dynamics simulations, due to the limited adsorption sites on the surface of C-S-H gels, the adsorption rate of Na^+^ decreases with increasing ion concentration. In contrast, the increase in sodium ion filling provides additional adsorption sites for chloride, leading to an increased adsorption rate of chloride ions [51]. On the other hand, the desorption of Ca^2+^ within the matrix causes the electric double-layer effect, making it difficult for Ca^2+^ in the solution to adsorb onto the matrix surface; Cl^−^ forms stable coordination with the matrix surface and the desorbed Ca^2+^, forming smaller ion clusters near the interface (Figure 3b). Furthermore, OH^−^ promotes the dissociation of silanol groups in C-S-H gel and adsorbs Ca^2+^ onto the gel surface, increasing the adsorption capacity for Cl^−^ [83]. Future research can further explore the impact of different combinations of cations and anions on the adsorption behavior of C-S-H gels, as well as how these interactions influence the macroscopic properties of the material.

### 3.3. Ca^2+^ in C-S-H Gel

**Figure 4:**
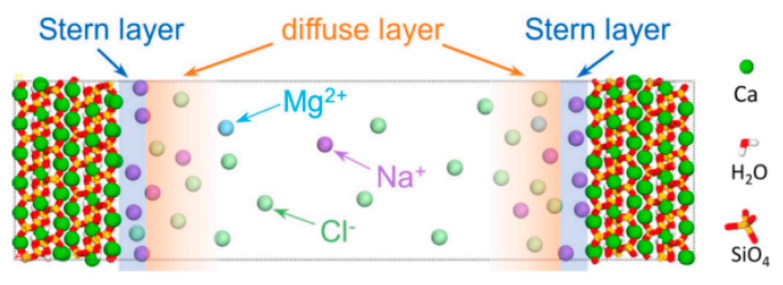
Double-layer effect [54].

Ca^2+^ in C-S-H gel includes both surface Ca^2+^ and constrained Ca^2+^ within the matrix. Surface Ca^2+^ experiences less constraint, diffuses more rapidly, and is more likely to desorb into the nanopore solution. Ca^2+^ enhances the interaction between adjacent silicate tetrahedra, which can increase the structural strength of the C-S-H gel. Decalcification helps to increase the average chain length of the C-S-H gels. It alters its pore structure, with most experimental studies attributing the acceleration of C-S-H gel decalcification to differences in solution pH and ionic solubility [84]. Molecular dynamics have revealed another mechanism for decalcification from the double-layer effect [54], where ions in seawater rapidly form a double-layer structure on the surface of C-S-H gel, as shown in Figure 4. Many cations rapidly adsorb onto the surface of C-S-H gel, forming a positively charged layer. This layer compensates for the negatively charged calcium vacancies by adsorbing cations such as Na^+^, leading to ion exchange that accelerates the dissolution of calcium. Furthermore, the C-S-H gels with a high Ca/Si ratio exhibit a lower dissolution-free energy, leading to more decalcification in the solution.

Studies on the adsorption of different ions (Na^+^, SO_4_^2−^, Mg^2+^, etc.) by C-S-H gel through experiments, nano-characterization techniques, and molecular dynamics have revealed that the adsorption mechanisms of these ions are related to the presence of Ca^2+^ [65,85,86].

(1) Na^+^: Na^+^ is connected to the non-bridging oxygen sites of the silicate chains on the surface of the C-S-H gel through Na–Os bonds. Due to the strong interaction between Na^+^ and the C-S-H gel interface lingers on the surface for a long time, causing surface Ca^2+^ to detach and form Ca–Cl ion clusters in the solution.

(2) SO_4_^2−^: With the increase in sulfate solution concentration, SO_4_^2−^ adsorption on the surface of C-S-H gel increases. SO_4_^2−^ is attracted by the electrostatic force between silicate oxygen atoms and Ca^2+^ on the solid surface. An increase in the calcium-to-silicate ratio in C-S-H gel is beneficial for the fixation of SO_4_^2−^ on the silicate surface. Molecular simulations have found that SO_4_^2−^ forms Ca–SO_4_ ion clusters with surface Ca^2+^, adsorbed on C-S-H gel, which hinders the interaction between Ca^2+^ and non-bridging oxygen on the silicate chains inside the C-S-H gel.

(3) Mg^2+^: Research using SEM, XRD, and other methods has studied the erosive effects of magnesium sulfate solutions on cement and found that Mg^2+^ causes decalcification, dissolution, and decomposition of C-S-H gel, enhancing the sulfate erosion effect. Magnesium ions enter the C-S-H gel, replacing Ca^2+^ to form M-S-H, leading to a complete loss of concrete strength. Magnesium ions enter the C-S-H gel, replacing Ca^2+^ to form M-S-H, leading to a complete loss of concrete strength. When Mg^2+^ erodes the silicate cement paste, it first reacts with OH^−^ in the pore solution to form insoluble Mg(OH)_2_, which fills the pores of the paste and hinders ion diffusion. It encapsulates the surface of the particles, delaying the hydration of the unhydrated cementitious material particles in the paste, resulting in a reduced degree of hydration of the cementitious paste. At the same time, the decrease in alkalinity leads to the decomposition of some C-S-H gel, which re-aggregates with Mg^2+^ to form M-S-H and amorphous hydrated silica, causing decalcification of C-S-H gel and an increase in the average chain length.

Ca^2+^ plays a crucial role in cross-linking within C-S-H gel and also influences the degree of polymerization of silicate tetrahedra within the C-S-H gel. Therefore, it is essential to investigate the interactions between Ca^2+^ and other ions from a molecular dynamics perspective. While some widely accepted conclusions already exist, the content and direction of research still require further refinement.

### 3.4. Transport Properties of Water Molecules and Ions

C-S-H gel is a porous gel system that includes capillary and gel pores, with numerous micropores ranging from 0.5 to 10 nm. These pores serve as transport channels for water molecules and ions within the gel. The transport performance of water molecules and ions in the nano-pores of C-S-H gels is closely related to the mechanical properties and durability of cement-based materials. The main modes of transport for water molecules and ions in the C-S-H gel pores are permeation, diffusion, and capillary action.

Water molecules at different locations within C-S-H gel exhibit varying diffusion coefficients due to differences in geometric constraints and bonding effects: capillary water molecules > surface water molecules > interlayer water molecules [87]. Ion transport within C-S-H gel pores is contingent upon water molecules. Molecular dynamics simulations by some scholars have investigated the transport characteristics of water molecules and ions in solutions, revealing that ions lag behind water molecules in migration rate within the C-S-H gel nanochannels [62,75]. Furthermore, the presence of positive and negative ions on the C-S-H gel surface, such as Cl^−^ and Ca^2+^, tend to aggregate into clusters, which accumulate in the nanochannels and reduce the diffusion rate of water molecules and the further penetration of other ions [88]. Tu et al. [89] simulated the transport of different ionic compounds within C-S-H gel nanochannels under paired coupling conditions, finding the transport rate of anions entering the C-S-H gel nanochannels to be SO_4_^2−^ > Cl^−^ > NO^2−^. Jiang et al. [90] discovered through simulation that the adsorption capacity of the C-S-H gel surface for monovalent cations increases with decreasing ionic radius, in the order Of Na^+^>> K^+^ > Cs^+^.

**Figure 5:**
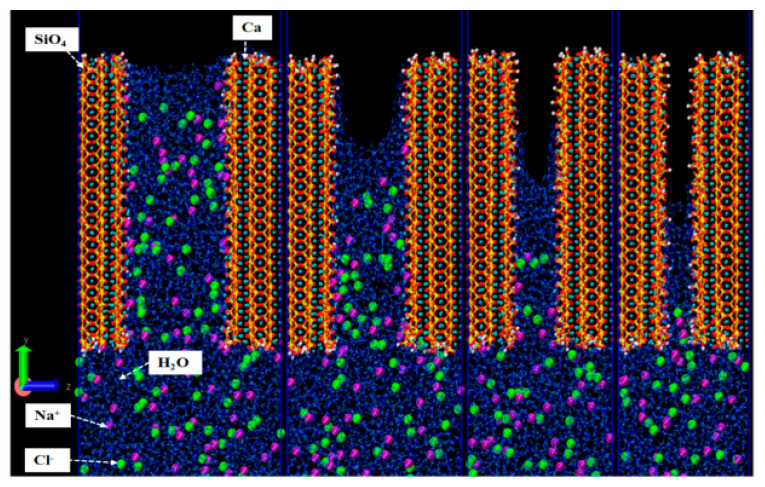
Capillary flow of water molecules, Na⁺, and Cl⁻ in pores of varying sizes in gel (from left to right, 3.5 nm, 2.5 nm, 1.5 nm, 1.0 nm) [62].

According to molecular dynamics simulation results, the transport of water molecules and ions in C-S-H gel is also influenced by factors such as the Ca/Si ratio, pore size, temperature, and concentration. Ions have a more significant diffusion coefficient in C-S-H gels with a low Ca/Si ratio [75]. Changes in the nanopore size of C-S-H gel significantly affect the arrangement of water molecules, particularly the structure of surface water molecules. Yang et al. [62] constructed four C-S-H gel models with gel pore sizes of 3.5 nm, 2.5 nm, 1.5 nm, and 1.0 nm in NaCl solution. As shown in Figure 5, when the pore size decreases from 3.5 nm to 1 nm, the penetration depth of the solution in the nanochannels decreases, and the transport velocity of Cl^−^ and Na^+^ is less than that of water molecules when the pore size is less than 2.5 nm. Correspondingly, as the nanopore size increases, the diffusion coefficients of water molecules and ions increase, and the penetration depth also increases [88].

## 4. Conclusions

Currently, most research is based on macroscopic experiments to study the impact of water molecules and ions on cement-based materials. Utilizing molecular simulation technology to investigate the adsorption and transport of water molecules and different ions in C-S-H gels from a mesoscale perspective has important theoretical significance and practical value. This review article is based on the current applications of molecular dynamics simulation methods in cement-based materials. It provides a systematic overview of the development of C-S-H gel models and the current state of molecular dynamics applications. Furthermore, it emphasizes the summarization and analysis of research advancements regarding the adsorption and transport characteristics of water molecules and ions within C-S-H gels. Although molecular dynamics research on the adsorption and transport of water molecules and ions in C-S-H gels has made preliminary progress, many deficiencies still need further strengthening. Mainly including the following:

(1) The amorphous structure of C-S-H gels is characterized by long-range order and short-range disorder, and there is a diversity in the selection of C-S-H gel models in molecular simulation processes, which cannot fully replace the natural C-S-H gel. There is a need for further exploration to construct a rational, universal, and accurate structural model of C-S-H gel. At the same time, cement-based materials are a very complex system, and the degree of cement hydration changes the cross-sectional shape and surface characteristics of the C-S-H gel pore channels. Focusing solely on C-S-H gel as the research object in the hydration products models is idealized and singular, and it will be possible in the future to consider modeling combinations of various hydration products to obtain data and conclusions that more accurately reflect the actual situation.

(2) Research on the adsorption and transport of water molecules and ions in C-S-H gels and their impact on the mechanical properties of cement-based materials is still in the theoretical analysis stage, and most studies lack corresponding experimental support. At the same time, relevant research is not detailed enough, such as the impact of environmental temperature, gel pore size, saturation degree, and other factors that have not yet reached a unified conclusion. In addition, studies on the adsorption and transport characteristics of aggressive ions in C-S-H gels are mostly single-ion erosion, and there is little research on the adsorption and transport under the interaction of multiple ions.

(3) With the widespread use of mineral admixtures in concrete, Al doping in C-S-H gel is becoming increasingly common. Although some scholars have compared the adsorption and transport characteristics of water and ions in C-S-H gel with those in calcium silicate aluminate hydrate (C-A-S-H) gel, research on the adsorption and transport of water molecules and ions in C-A-S-H is not yet in-depth and deserves further systematic study.

## Figures and Tables

**Table 1:** Comparison and analysis of C-S-H gel structure models.

Model Type	Theoretical Basis	Structural Foundation	Structural Shape	Application Scope
Taylor model	Crystal theory	Tobermorite, Jennite	Layered and fragmented	Pore structure, Ca/Si ratio, mechanical properties, etc.
Solid Solution	Thermodynamic theory	Tobermorite, Ca(OH)_2_	Layered	Cement hydration, solving thermodynamic quantitative problems, etc.
R–G model	Thermodynamic theory	Tobermorite, Jennite, Ca(OH)_2_	Layered	Ca/Si ratio, water content, average silicon chain length, etc.

**Table 2:** Lattice parameters of Tobermorite and Jennite.

Crystal Structure	Tobermorite-9Å	Tobermorite-11Å	Hamid Tobermorite-11Å	Tobermorite-14Å	Jennite
space group	C-1	Bm	P21	B11b	P-1
Lattice Type	triclinic	monoclinic	monoclinic	monoclinic	triclinic
a/Å	11.156	6.732	6.69	6.735	10.576
b/Å	7.303	7.369	7.39	7.425	7.265
c/Å	9.566	22.68	22.77	27.989	10.931
α/(°)	101.08	90	90	90	101.3
β/(°)	92.83	90	90	90	96.98
γ/(°)	89.98	123.18	123.49	123.25	109.65

## Data Availability

The original contributions presented in the study are included in the article, further inquiries can be directed to the corresponding author.
